# Impact of Health Facility-Based Insecticide Treated Bednet Distribution in Malawi: Progress and Challenges towards Achieving Universal Coverage

**DOI:** 10.1371/journal.pone.0021995

**Published:** 2011-07-21

**Authors:** Jacek Skarbinski, Dyson Mwandama, Madalitso Luka, James Jafali, Adam Wolkon, David Townes, Carl Campbell, John Zoya, Doreen Ali, Don P. Mathanga

**Affiliations:** 1 Malaria Branch, Centers for Disease Control and Prevention, Atlanta, Georgia, United States of America; 2 Malaria Alert Centre, University of Malawi College of Medicine, Blantyre, Malawi; 3 Epidemic Intelligence Service, Centers for Disease Control and Prevention, Atlanta, Georgia, United States of America; 4 Division of Emergency Medicine, University of Washington, Seattle, Washington, United States of America; 5 Center for Tropical and Global Emerging Diseases, University of Georgia, Athens, Georgia, United States of America; 6 National Malaria Control Programme, Ministry of Health, Lilongwe, Malawi; 7 Department of Community Health, University of Malawi College of Medicine, Blantyre, Malawi; Kenya Medical Research Institute - Wellcome Trust Research Programme, Kenya

## Abstract

**Background:**

High levels of insecticide treated bednet (ITN) use reduce malaria burden in countries with intense transmission such as Malawi. Since 2007 Malawi has implemented free health facility-based ITN distribution for pregnant women and children <5 years old (under-5s). We evaluated the progress of this targeted approach toward achieving universal ITN coverage.

**Methods:**

We conducted a cross-sectional household survey in eight districts in April 2009. We assessed household ITN possession, ITN use by all household members, and *P. falciparum* asexual parasitemia and anemia (hemoglobin <11 grams/deciliter) in under-5s.

**Results:**

We surveyed 7,407 households containing 29,806 persons. Fifty-nine percent of all households (95% confidence interval [95% CI]: 56–62), 67% (95% CI: 64–70) of eligible households (i.e., households with pregnant women or under-5s), and 40% (95% CI: 36–45) of ineligible households owned an ITN. In households with at least one ITN, 76% (95% CI: 74–78) of all household members, 88% (95% CI: 87–90) of under-5s and 90% (95% CI: 85–94) of pregnant women used an ITN the previous night. Of 6,677 ITNs, 92% (95% CI: 90–94) were used the previous night with a mean of 2.4 persons sleeping under each ITN. In multivariable models adjusting for district, socioeconomic status and indoor residual spraying use, ITN use by under-5s was associated with a significant reduction in asexual parasitemia (adjusted odds ratio (aOR) 0.79; 95% CI: 0.64–0.98; p-value 0.03) and anemia (aOR 0.79; 95% CI 0.62–0.99; p-value 0.04). Of potential targeted and non-targeted mass distribution strategies, a campaign distributing 1 ITN per household might increase coverage to 2.1 household members per ITN, and thus achieve near universal coverage often defined as 2 household members per ITN.

**Conclusions:**

Malawi has substantially increased ITN coverage using health facility-based distribution targeting pregnant women and under-5s, but needs to supplement these activities with non-targeted mass distribution campaigns to achieve universal coverage and maximum public health impact.

## Introduction

Insecticide treated bednets (ITNs) have been shown to reduce malaria-associated morbidity and mortality [1]. However, ITN coverage measured as either household possession or use the previous night continues to be suboptimal in most of sub-Saharan Africa. Increasing ITN coverage has been done using a variety of distribution strategies including, the development of commercial markets, social marketing interventions, and distribution through health facilities, community groups and mass distribution campaigns [2], [3], [4], [5], [6], [7], [8], [9], [10], [11], [12], [13]. All of these strategies can contribute to both ‘catch up’ and ‘keep up’ to increase ITN coverage [14], [15]. Currently, many countries are transitioning from targeted coverage of vulnerable groups such as children <5 years old and pregnant women, towards universal coverage as a means to maximally impact malaria transmission as has been suggested by prior studies [16], [17], mathematical models [18] and global policy [19].

Malawi distributes ITNs through three main mechanisms: 1) routine free distribution of ITNs for children born in health facilities, children attending their first visit under the Expanded Program on Immunization (EPI) if an ITN was not received at birth, and pregnant women at their first visit to an antenatal care (ANC) clinic; 2) periodic mass campaigns targeted at households in ‘hard to reach areas’; 3) traditional social marketing through private sector outlets. The United States President's Malaria Initiative (PMI) and the Global Fund are the main funders of ITNs in Malawi. Between 2007 and 2009, PMI and the Global Fund have purchased approximately 4.5 million ITNs, the majority of which have been distributed through health facilities to pregnant women and children <5 years old [20]. In addition, 1.1 million ITNs have been distributed through a sub-national campaign targeting rural ‘hard to reach areas’ in 2008. Between 2007 and 2009 approximately 4.5 million ITNs have been distributed in a country of 14.1 million people, or roughly 0.33 ITNs per person.

In April 2009 as part of an annual household survey, we assessed household ITN possession and use in eight sentinel districts across Malawi. As Malawi aims to achieve universal coverage with ITNs, we assessed the progress and challenges towards achieving universal coverage.

## Methods

### Ethics statement

This study was approved by the University of Malawi College of Medicine ethical committee and the Centers for Disease Control and Prevention Institutional Review Board. Written informed consent was obtained from all adult participants and all parents or guardians of children.

### Household survey

**Figure 1:**
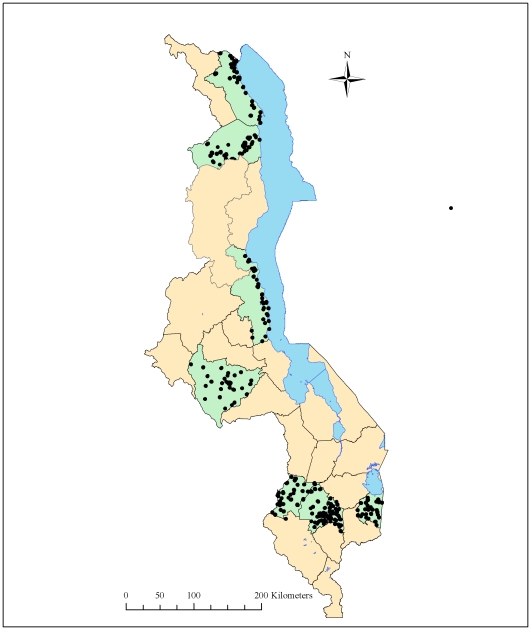
Districts and census enumeration areas included in the household survey in eight districts, Malawi 2009. Districts included in the household survey are shaded green and sampled census enumeration areas are represented by black circles.

We conducted a cross-sectional household survey from April 16–30, 2009 at the end of the long rains and in the middle of the high malaria transmission season. The study was conducted in both urban and rural communities in eight districts in Malawi (Lilongwe, Blantyre, Mwanza, Chiradzulu, Phalombe, Rumphi, Nkhotakota, and Karonga), which contain approximately 33% of the entire population of Malawi and are approximately evenly divided between the north, central and south regions (Figure 1). The districts were purposively selected in collaboration with the Ministry of Health to serve as sentinel districts to monitor malaria burden and coverage of malaria control interventions.

We used a two-stage cluster sampling design. The first stage was composed of selecting enumeration areas (EAs). Altogether, 30 EAs per district were chosen using systematic random sampling with selection probability proportional to estimated size using the 1998 census. In the second stage, we divided the EA into segments of roughly 30–60 households and randomly selected a segment using a personal digital assistant (PDA; Dell Axim X50s, Dell Inc., Austin, TX, USA) with a specially designed program for random segment selection developed by the Centers for Disease Control and Prevention, USA. All households or a randomly selected subset of households in a selected segment were invited to participate in the survey. Informed consent was obtained from the head of household to participate in the survey. All household members were asked to participate and those who agreed were asked standardized questions. Household members were defined as all persons who usually live in the household as well as guests of the household who stayed in the household last night. If family members in a household were not home, the household was revisited at the end of the day. If no one was available after two visits, the household was dropped from the survey; these households were not replaced. Information on bednets was collected by creating a bednet roster. Bednets in the household were directly observed and classified as long-lasting insecticidal net using identifying information such as labels or characteristic features. For any bednet that could not be visualized or could not be clearly identified, the interviewer asked about treatment with insecticide either at the factory or at home. After completing a roster of all bednets in the household, a roster of all household members was created. We recorded which household member slept under which bednet allowing us to collected detailed, linked information on household members and bednets.

All data were collected electronically using a questionnaire designed and programmed into PDAs using Visual CE 11.0 (Syware Inc, Cambridge, MA, USA) [21]. The questionnaire was designed in English and translated into the three main languages spoken in the selected districts, namely Chichewa, Chitumbuka and Chiyao. All questions were closed-ended, but the choices were not read to respondents. Responses were coded as ‘other’ if the respondent's answer did not match any of the anticipated categories. Skip patterns, internal logic checks, and informational pop-up screens were programmed into the PDA-based survey to improve the ease and accuracy of data collection.

### Laboratory procedures and treatment

Parent or guardian consent was obtained for a finger prick blood sample for all children <5 years old. A thick blood film was prepared and hemoglobin concentration measured using the Hemocue Hb 201+ Analyzer (Hemocue Inc, Cypress, CA, USA). The thick blood smears were stained with Field's Stain A and B (azure dye and eosin). Parasite densities were calculated by counting the number of asexual stage parasites per 200 white blood cells (WBCs), assuming 8,000 WBCs/deciliter of blood. Blood smears were considered negative if no parasites were found after counting 200 fields. Thick films were examined at central laboratory facilities located in each region. For quality control purposes, 10% of slides were re-examined by an expert microscopist at a reference laboratory in Blantyre, Malawi.

Hemoglobin results were shared with the parent or guardian at the time of the household visit. Children with hemoglobin levels <8 g/dl were provided written results, given artemether-lumefantrine, albendazole (if >24 months of age), an age appropriate two-week dosage of daily iron and referred to a health facility. Children with a history of fever received immediate presumptive treatment for malaria using artemether-lumefantrine, according to Malawi national treatment guidelines. Children who were treated with artemether-lumefantrine within the past two weeks, but remained febrile at the time of the survey were treated with quinine. Children who were found to be seriously ill, as determined by the survey nurses, were provided transportation to the nearest health facility.

### Definitions

An ITN was defined as any long-lasting insecticidal net, any bednet factory-treated with insecticide and obtained less than 12 months ago, or any bednet treated with insecticide less than 12 months ago. Bednet and ITN use were defined as reportedly sleeping under a bednet or ITN the previous night, respectively. Households were considered eligible for health facility-based ITN distribution if they contained a pregnant woman or child <5 years old at the time of the survey. In children <5 years old, anemia was defined as hemoglobin <11 gm/dl. Parasitemia was defined as presence of asexual *P.falciparum* parasites on a thick blood film.

### Data analysis

All responses were entered directly into a PDA database in the field. Data were downloaded into a relational database using Access 2000 software (Microsoft Inc., Redmond, WA, USA). Analyses were performed using SAS version 9.2 (SAS Institute, Cary, NC, USA) using the proc survey procedures, which uses the Taylor expansion method to account for cluster sampling and unequal selection probabilities. Analyses were weighted, and weights equaled the inverse of the exact probability of selection. Percentages reported in this report reflect this weighting unless otherwise noted. Statistical significance was defined as a p-value≤0.05.

A relative index of household socioeconomic status (SES) was derived based on 19 categorical variables using principal components analysis (PCA) [22], [23]. This technique generates an index providing maximum discrimination between households, which is normally distributed with a mean of zero and standard deviation of one. The variables included were a combination of utilities (sources of water, light, toilet type), use of domestic workers, and ownership of assets (land, bicycle, motorcycle, car, oxcart, lamp, radio, television, telephone, cell phone, refrigerator, bed, sofa, table). House construction variables (floor, walls and roofs) were not used as they might be independently associated with outcomes of interest such as parasitemia or anemia in children <5 years old. The index was calculated across all surveyed households. The first principal component explained 22% of the variability in the SES variables, a similar proportion to that explained in other such analyses [22], [23], [24], [25], [26]. The assets which made the greatest change to the PCA score were ownership of a sofa, bed or television and availability of electricity. The PCA scores ranged from −13.0764 to 2.06105. Households were classified on the basis of their PCA score into SES quintiles, with mean PCA scores of −3.20, −0.10, 0.70, 1.09, and 1.58 from least poor to poorest, respectively. Principal components analysis scores were not adjusted for household size, as the benefits of household construction, utilities and many durable assets were assumed to be available to all members of a given household [23]. Average household size was slightly higher among households of higher SES, meaning that, although households were equally distributed between SES quintiles by definition, 26% of individuals interviewed were in the least poor quintile, compared with 20% in the poorest quintile.

We used multivariate logistic regression to assess predictors of household ITN possession, use of ITNs by persons who reside in households with at least one ITN, and parasitemia and anemia in children <5 years old. All multivariate models included district and SES by wealth quintile as co-variates in addition to the variables of interest.

### Quantification of potential coverage achievable by different distribution campaigns

We quantified the potential coverage that could be achieved with different types of distribution campaigns with a focus on ITN ownership by households with and without traditional target groups. Using data on household composition regarding the number and type of household members, we quantified the inputs needed for and the ITN coverage that could maximally be achieved by a ‘perfect’ campaign either targeting a particular population (children <5 years old or children 5–15 years old) or providing universal coverage with either 1 ITN per household, 2 ITNs per household, 1 ITN per sleeping space, or 1 ITN per 2 household members. Our analysis assumed 100% coverage of the target population and quantified the theoretical inputs in ITNs required to cover the population in the eight districts. The quantification of ITNs needed for each strategy did not account for wastage, inaccurate census denominators, buffer stocks, ITN degradation and other factors that need to be considered in the actual planning of a distribution campaign and are well described elsewhere [27]. All of these factors should similarly bias upwards the estimates for ITN needs for all campaign distribution strategies.

## Results

We surveyed 30 EAs per district, 240 EAs in total, and collected data on 7,407 households containing 29,806 household members (7,504 children <5 years old, 634 pregnant women, 6,783 children 5–15 years old, and 14,885 non pregnant adults >15 years old) and 8,141 untreated bednets and ITNs. Mean household size and mean number of sleeping spaces varied by district with the mean household size of 4.0 household members and a mean number of sleeping spaces per household of 1.9 (Table 1). In addition, 70% of households had at least one child <5 years old, 9% had at least one pregnant woman and 72% had at least one child <5 years old or a pregnant woman.

**Table 1:** Household characteristics in eight districts, Malawi, 2009 (N = 7,407).

	Blantyre (N = 795)	Chiradzulu (N = 945)	Karonga (N = 929)	Lilongwe (N = 922)	Mwanza (N = 1,125)	Nkhotakhota (N = 884)	Phalombe (N = 904)	Rumphi (N = 903)	Total (N = 7,407)
	% (95%CI)^b^	% (95%CI)^b^	% (95%CI)^b^	% (95%CI)^b^	% (95%CI)^b^	% (95%CI)^b^	% (95%CI)^b^	% (95%CI)^b^	% (95%CI)^b^
**District population** a	999,491	290,946	272,789	1,897,167	94,476	301,868	313,227	169,112	4,339,076
**Mean household size**	4.0 (3.8–4.2)	3.6 (3.5–3.8)	4.2 (4.0–4.5)	4.0 (3.8–4.3)	4.2 (4.0–4.4)	4.4 (4.1–4.7)	3.7 (3.5–3.8)	4.1 (3.9–4.3)	4.0 (3.9–4.1)
**Mean number of sleeping space per household**	1.9 (1.7–2.0)	3.4 (2.8–4.0)	2.1 (2.0–2.3)	1.6 (1.5–1.7)	1.8 (1.7–1.9)	2.1 (2.0–2.2)	1.9 (1.8–2.0)	2.0 (1.8–2.1)	1.9 (1.8–2.0)
**Households eligible for health facility-based insecticide treated bednet distribution**									
Households with at least one child <5 years old	70% (66–74)	72% (66–77)	60% (56–63)	68% (61–74)	69% (65–72)	70% (67–74)	75% (68–81)	88% (82–93)	70% (67–72)
Households with at least one pregnant woman	9% (7–11)	6% (5–7)	5% (4–7)	10% (8–12)	8% (6–10)	9% (7–11)	9% (7–11)	13% (10–16)	9% (8–10)
Households with at least one child <5 years old or pregnant woman	74% (70–77)	73% (68–79)	62% (58–65)	71% (64–77)	71% (68–74)	72% (69–76)	78% (72–84)	89% (84–94)	72% (70–75)

a Data from 2008 Malawi census.

b 95% confidence interval.

### Household bednet and ITN possession

In the eight surveyed districts, 68% of all households owned at least one bednet, 59% owned at least one ITN, and 57% owned at least one ITN obtained through health facility-based distribution (Table 2). Household ITN possession was higher in households that had at least one child <5 years old or a pregnant woman (73%; 95% confidence interval (95%CI): 70–75) than households who had neither (55%; 95%CI: 50–60). Thirty six percent (36%) of households ineligible for health facility-based distribution still owned at least one ITN obtained from a health facility. The mean number of ITNs was higher (0.96) in households eligible for health facility-based distribution than in households who were ineligible for health facility-based distribution (0.60). Few households owned ITNs obtained from the free distribution campaign (2%) or purchased in the market (2%).

**Table 2:** Household bednet and insecticide treated bednet (ITN) ownership in eight districts, Malawi, 2009 (N = 7,407).

		Households eligible for health facility-based ITN distribution			
	All households (N = 7,407)	Households with at least one child <5 years old (N = 5,266)	Households with at least one pregnant woman (N = 631)	Households with at least one child <5 years old or pregnant woman (N = 5,438)	Households ineligible for health facility- based ITN distribution (N = 1,969)
	% (95%CI)^a^	% (95%CI)^a^	% (95%CI)^a^	% (95%CI)^a^	% (95%CI)^a^
**Any bednet**	68% (65–70)	73% (70–75)	69% (64–74)	73% (70–75)	55% (50–60)
**Any ITN**	59% (56–62)	67% (64–70)	60% (53–66)	67% (64–70)	40% (36–45)
ITN obtained from a health facility	57% (54–59)	65% (62–68)	58% (52–64)	64% (61–67)	36% (32–40)
ITN obtained from a campaign	2% (2–3)	2% (2–3)	3% (1–5)	2% (2–3)	2% (1–3)
ITN purchased in market	2% (1–3)	1% (1–2)	1% (0–2)	1% (1–2)	4% (2–6)
**Mean number of ITNs per household**	0.86 (0.80–0.91)	0.97 (0.91–1.02)	0.83 (0.74–0.93)	0.96 (0.90–1.01)	0.60 (0.53–0.68)
**Mean number of ITNs per household member**	0.22 (0.20–0.23)	0.22 (0.22–0.24)	0.21 (0.19–0.24)	0.23 (0.21–0.24)	0.18 (0.16–0.21)

a 95% confidence interval.

We assessed predictors of household ITN possession using a multivariable logistic regression model (Table 3). Households in the poorest quintile were less likely to own an ITN than households in the least poor quintile (adjusted odds ratio (aOR) 0.62; 95%CI: 0.43–0.90; p-value 0.01). Households with pregnant women or children <5 years old (i.e. eligible for health facility-based distribution) were more likely to own ITNs (aOR 3.09; 95%CI: 2.57–3.7; p-value<0.001) than households who were ineligible for health facility-based distribution.

**Table 3:** Factors associated with household insecticide treated bednet (ITN) ownership in eight districts, Malawi, 2009 (N = 7,407).

Variable	Household ITN ownership	Adjusted odds ratio	
	n/N (%; 95%CI^a^)	(95%CI^a^)	p-value
**District**			
Blantyre	512/795 (64%; 60–69)	Referent	Referent
Chiradzulu	549/945 (58%; 53–63)	0.78 (0.59–1.05)	0.10
Karonga	581/929 (63%; 58–67)	1.06 (0.79–1.42)	0.69
Lilongwe	538/922 (58%; 51–65)	0.87 (0.60–1.27)	0.47
Mwanza	730/1125 (65%; 61–69)	1.21 (0.91–1.61)	0.18
Nkhotakhota	437/884 (50%; 45–54)	0.60 (0.46–0.78)	<0.001
Phalombe	503/904 (55%; 51–60)	0.71 (0.52–0.97)	0.03
Rumphi	510/903 (57%; 50–63)	0.59 (0.42–0.82)	0.002
**Socioeconomic status**			
Poorest	707/1478 (51%; 46–56)	0.62 (0.43–0.90)	0.01
Second	691/1304 (55%; 51–59)	0.67 (0.48–0.93)	0.02
Third	970/1616 (62%; 57–67)	0.90 (0.64–1.28)	0.56
Fourth	1011/1528 (66%; 62–71)	1.12 (0.81–1.55)	0.51
Least poor	981/1481 (62%; 56–69)	Referent	Referent
**Household eligible for health facility-based ITN distribution**			
Eligible	3612/5438 (67%; 64–70)	3.09 (2.57–3.71)	<0.001
Ineligible	748/1969 (40%; 36–45)	Referent	Referent

a 95% confidence interval.

### Bednet and ITN use

Although overall bednet (58%) and ITN (49%) use among all persons in the survey was moderate, use among persons who resided in households with at least one ITN was high with 76% of all such persons sleeping under an ITN the previous night (Table 4). Use was even higher for populations targeted for health facility-based distribution with 88% of children <5 years old and 90% of pregnant women residing in a household with at least one ITN sleeping under an ITN the previous night. ITN use the previous night was lowest among non-pregnant persons 5–15 years old (36%). Of 8141 bednets, including 6,677 ITNs and 1,464 untreated bednets, registered in the survey, 98% (95%CI: 98–99) were directly visualized, and 92% (95%CI: 90–94) of ITNs and 94% (95%CI: 90–98) of untreated bednets were hanging at the time of the survey. Of the 6,677 ITNs in the survey, 94% (95%CI: 93–96) were long-lasting insecticidal nets. In addition, most ITNs were used by more than one person with a mean of 2.4 persons sleeping under each ITN.

**Table 4:** Insecticide treated bednet (ITN) use by persons who reside in any household and in a household that owns an ITN in eight districts, Malawi, 2009 (N = 29,806).

	All individuals	Children <5 years old	Pregnant women	Non-pregnant persons 5–15 years old	Non-pregnant persons >15 years old
	% (95%CI)^a^	% (95%CI)^a^	% (95%CI)^a^	% (95%CI)^a^	% (95%CI)^a^
**Resides in any household**	**(N = 29,806)**	**(N = 7,504)**	**(N = 634)**	**(N = 6,783)**	**(N = 14,885)**
**Uses any bednet**	58% (55–61)	68% (65–71)	66% (60–71)	44% (40–48)	56% (56–62)
**Uses any ITN**	49% (45–51)	61% (58–64)	54% (47–60)	36% (33–40)	48% (44–51)
ITN obtained from health facility	46% (43–49)	58% (55–61)	52% (45–58)	34% (31–38)	45% (42–48)
ITN obtained from campaign	1% (1-1)	1% (1-2)	1% (0-3)	1% (0-1)	1% (1-1)
ITN purchased in market	1% (1-2)	1% (0-1)	<1% (0-1)	1% (1-1)	1% (1-2)
**Resides in a household that owns an ITN**	**(N = 18,967)**	**(N = 5,082)**	**(N = 414)**	**(N = 4,428)**	**(N = 9,043)**
**Uses any bednet**	81% (79–83)	92% (90–94)	95% (93–98)	61% (58–65)	84% (82–86)
**Uses any ITN**	76% (74–78)	88% (87–90)	90% (85–94)	55% (52–59)	78% (76–81)
ITN obtained from health facility	72% (69–75)	85% (82–87)	86% (81–92)	53% (49–56)	74% (71–77)
ITN obtained from campaign	2% (1-2)	2% (1-3)	2% (0-4)	1% (1-2)	2% (1-2)
ITN purchased in market	2% (1-2)	1% (1-2)	<1% (0-1)	1% (1-2)	2% (1-3)

a 95% confidence interval.

In a multivariable logistic regression model, ITN use by persons who resided in a household with at least one ITN was associated with socioeconomic status (persons in poorest households were significantly more likely to use ITNs than persons in least poor households), age group, pregnancy status (children <5 years old and pregnant women were significantly more likely to use ITNs while non-pregnant children 5–15 years old were significantly less likely to use ITNs than non-pregnant persons >15 years old), and number of ITNs per household (increased use in households with more ITNs per household) (Table 5).

**Table 5:** Factors associated with insecticide treated bednet (ITN) use by person who reside in households with at least one ITN in eight districts, Malawi, 2009 (N = 18,967).

Variable	ITN use	Adjusted odds ratio	
	n/N (%; 95%CI^a^)	(95%CI^a^)	p-value
**District**			
Blantyre	1743/2209 (79%; 75–82)	Referent	Referent
Chiradzulu	1688/2142 (79%; 75–82)	0.85 (0.64–1.13)	0.26
Karonga	1906/2657 (71%; 67–76)	0.46 (0.35–0.61)	<0.001
Lilongwe	1693/2293 (74%; 69–79)	0.68 (0.48–0.97)	0.03
Mwanza	2583/3302 (78%; 74–82)	0.77 (0.56–1.06)	0.11
Nkhotakhota	1422/2118 (67%; 62–71)	0.45 (0.34–0.61)	<0.001
Phalombe	1635/2015 (81%; 78–84)	0.89 (0.66–1.19)	0.42
Rumphi	1801/2231 (80%; 75–85)	0.84 (0.56–1.27)	0.41
**Socioeconomic status by asset index**			
Poorest	2232/2817 (78%; 66–74)	2.04 (1.45–2.88)	<0.001
Second	2222/2825 (79%; 77–83)	2.15 (1.61–2.87)	<0.001
Third	3152/4116 (78%; 74–82)	1.89 (1.41–2.53)	<0.001
Fourth	3496/4531 (78%; 74–81)	1.72 (1.28–2.31)	<0.001
Least poor	3369/4678 (70%; 66–74)	Referent	Referent
**Target groups**			
Child <5 years old	4516/5082 (88%; 87–90)	2.18 (1.90–2.50)	<0.001
Pregnant woman	367/414 (90%; 85–94)	2.56 (1.58–4.15)	<0.001
Non-pregnant children 5–15 years old	2496/4428 (55%; 52–59)	0.28 (0.24–0.34)	<0.001
Non-pregnant persons >15 years old	7092/9043 (78%; 76–81)	Referent	Referent
**Number of ITNs per household (per additional ITN)**	Mean 1.45 (95%CI: 1.40–1.50)	2.40 (2.05–2.80)	<0.001

a 95% confidence interval.

### Strategies to increase household ITN possession

**Figure 2:**
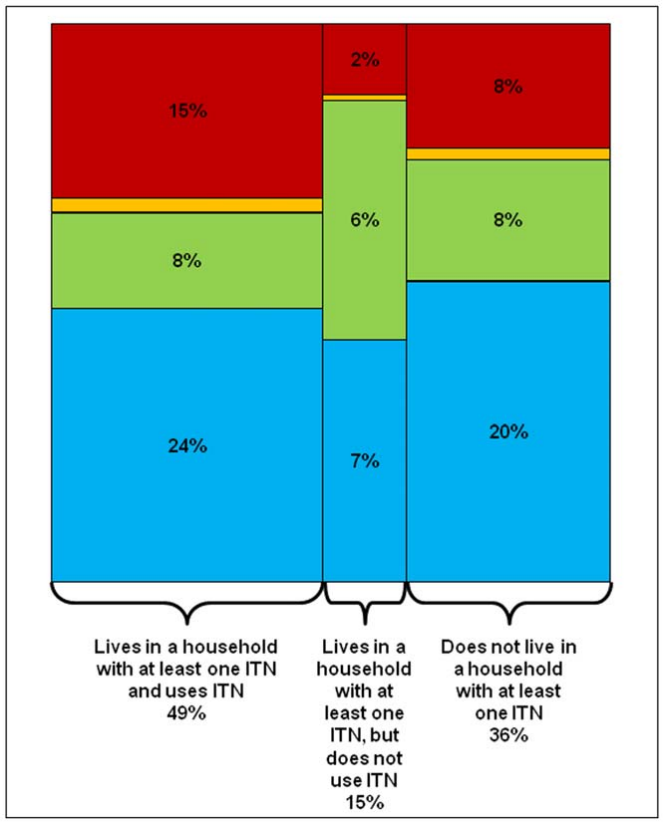
Relationship between household insecticide treated bednet (ITN) possession and use by all household members by age group and pregnancy status in eight districts, Malawi, 2009 (N = 29,806). Red = children <5 years old (25% of population). Orange = pregnant women (2% of population). Green = children 5–15 years old (23% of population). Blue = Non-pregnant persons >15 years old (50% of population).

Currently, Malawi relies on a strong health facility-based distribution system with some supplemental ITN distribution via social marketing and targeted campaigns. The current distribution system has led to 49% of all persons residing in a household with at least one ITN having used an ITN the previous night (Figure 2). However, 15% of the population resides in a household with at least one ITN, but did not use an ITN the night before; these persons, most often children 5–15 years, likely do not use an ITN as there are insufficient ITNs for all household members in their household. In addition, 36% of the population resides in a household that does not own at least one ITN. In order to achieve universal coverage, these gaps need to be addressed [28].

Using this survey, we estimate that there are 932,597 ITNs in the eight survey districts with 64% of persons residing in a household with at least one ITN and a mean number of 0.21 ITNs per household member. Either targeted or universal coverage campaigns are needed to supplement health facility-based distribution. We assessed the potential coverage that could be achieved with different types of distribution campaigns (Table 6). Targeted campaigns aimed at providing an ITN to every child <5 years old or every child 5–15 years old would achieve only moderate household coverage with 86% and 84% of households owning at least one ITN after the campaign, respectively. In addition, these targeted distribution strategies would require similar inputs as a universal coverage campaign distributing 1 ITN per household (approximately 1 million ITNs for a population of 4.3 million persons). All universal coverage campaigns would theoretically achieve 100% household ITN possession. A campaign that would deliver 1 ITN per household would require about 1 million ITNs, campaigns that deliver 2 ITNs per household or 1 ITN per sleeping space would require about 2 million ITNs each, and a campaign that delivers 1 ITN per 2 persons would require about 3 million ITNs. The mean number of ITNs per household member would increase from 0.47 in a campaign that delivers 1 ITN per household to 0.97 in a campaign that delivers 1 ITN per 2 persons. Campaigns that deliver either 2 ITNs per household or 1 ITN per sleeping space are roughly equivalent in terms of coverage achieved (100% of household would own at least one ITN and approximately 0.7 ITNs per household member).

**Table 6:** Estimated household insecticide treated bednet (ITN) possession using different campaign-based distribution strategies in eight districts, Malawi, 2009.

		Targeted campaigns		Universal coverage campaigns			
	ITN possession in 2009	1 ITN per <5 year old	1 ITN per 5–15 year old	1 ITN per household	2 ITNs per household	1 ITN per sleeping space^b^	1 ITN per 2 persons
**Number of ITNs required**	932,597^a^	1,092,412	987,451	1,086,236	2,172,472	2,075,285	3,276,002^c^
**Resides in a household with at least one ITN**							
All individuals	64%	86%	84%	100%	100%	100%	100%
Children <5 years old	68%	100%	83%	100%	100%	100%	100%
Pregnant women	65%	85%	82%	100%	100%	100%	100%
Non-pregnant persons 5–15 years old	65%	87%	100%	100%	100%	100%	100%
Non-pregnant persons >15 years old	61%	83%	80%	100%	100%	100%	100%
**Mean number of ITNs per household**	0.86	1.86	1.77	1.86	2.86	2.77	3.87
**Mean number of ITNs per household member**	0.21	0.47	0.44	0.47	0.72	0.69	0.97

Note: Based on total population of 4,339,876 persons in the eight surveyed districts according to the 2008 Malawi census.

a Estimated number of ITNs currently in the eight surveyed districts.

b Estimated 1.91 sleeping spaces per household in the eight surveyed districts.

c In households with an odd number of household members, number of ITNs distributed is equal to number of household members divided by two plus one additional ITN (e.g. a household with 5 persons will receive 3 ITNs).

### Impact of ITN use on asexual parasitemia and anemia prevalence in children <5 years old

We assessed asexual parasitemia and anemia prevalence in children <5 years old. Parasitemia and anemia prevalence varied significantly between districts with the highest parasitemia prevalence in Phalombe (50%), the highest anemia prevalence in Nkhotakota (64%) and the lowest prevalence of both parameters in Rumphi District (5%) (Table 7, Table 8). In addition, children <5 years old residing in households in the poorer quintiles were more likely to be parasitemic and anemic than children residing in households in the least poor quintile. ITN use was associated with significantly lower parasitemia (aOR 0.79; 95%CI: 0.64–0.98; p-value 0.03) and anemia prevalence (aOR 0.79; 95%CI: 0.62–0.99; p-value 0.04) after adjusting for district, socioeconomic status and use of indoor residual spraying.

**Table 7:** Factors associated with asexual parasitemia in children <5 years old in eight districts, Malawi, 2009 (N = 6,581).

Variable	Asexual parasitemia prevalence	Adjusted odds ratio	
	n/N (%; 95%CI^a^)	(95%CI^a^)	p-value
**District**			
Blantyre	83/671 (12%; 7–18)	Referent	Referent
Chiradzulu	174/827 (21%; 17–24)	1.50 (0.88–2.56)	0.14
Karonga	30/578 (5%; 3–8)	0.40 (0.20–0.79)	0.008
Lilongwe	246/805 (30%; 24–36)	2.44 (1.41–4.24)	0.002
Mwanza	243/904 (27%; 22–32)	2.07 (1.18–3.63)	0.01
Nkhotakhota	282/849 (33%; 27–40)	3.38 (1.92–5.97)	<0.001
Phalombe	413/841 (50%; 43–56)	5.37 (3.08–9.39)	<0.001
Rumphi	56/1106 (5%; 3–7)	0.34 (0.18–0.64)	<0.001
**Socioeconomic status by asset index**			
Poorest	395/1231 (35%; 30–41)	3.46 (2.30–5.21)	<0.001
Second	317/1209 (29%; 26–33)	2.84 (2.03–3.97)	<0.001
Third	414/1540 (28%; 24–32)	2.64 (1.80–3.87)	<0.001
Fourth	285/1386 (21%; 17–25)	2.10 (1.45–3.05)	<0.001
Least poor	116/1215 (10%; 7–13)	Referent	Referent
**Bednet use**			
Used an ITN the previous night	883/3956 (23%; 20–27)	0.79 (0.64–0.98)	0.03
Used an untreated net the previous night	84/401 (15%; 10–20)	0.64 (0.43–0.96)	0.03
Did not use a net	560/2131 (29%; 25–34)	Referent	Referent
**IRS use**			
Lives in a house that received IRS	62/286 (21%; 15–28)	0.54 (0.37–0.80)	0.002
No IRS	1465/6295 (25%; 21–28)	Referent	Referent

a 95% confidence interval.

**Table 8:** Factors associated with any anemia (hemoglobin <11 gm/dl) in children <5 years old in eight districts, Malawi, 2009 (N = 6,818).

Variable	Anemia prevalence	Adjusted odds ratio	
	n/N (%; 95%CI^a^)	(95%CI^a^)	p-value
**District**			
Blantyre	332/674 (49%; 42–56)	Referent	Referent
Chiradzulu	422/834 (50%; 43–57)	0.89 (0.61–1.30)	0.55
Karonga	373/662 (58%; 48–68)	1.48 (0.93–2.36)	0.10
Lilongwe	516/806 (63%; 53–73)	1.53 (0.92–2.55)	0.10
Mwanza	595/977 (61%; 55–68)	1.37 (0.93–2.03)	0.11
Nkhotakhota	530/835 (64%; 57–69)	1.96 (1.27–3.02)	0.002
Phalombe	528/900 (59%; 53–65)	1.22 (0.84–1.76)	0.31
Rumphi	362/1130 (32%; 27–37)	0.45 (0.32–0.64)	<0.001
**Socioeconomic status by asset index**			
Poorest	801/1287 (65%; 58–72)	1.74 (1.13–2.68)	0.01
Second	729/1239 (61%; 56–67)	1.66 (1.18–2.32)	0.004
Third	878/1605 (59%; 54–65)	1.57 (1.11–2.22)	0.01
Fourth	725/1434 (53%; 47–59)	1.30 (0.94–1.82)	0.12
Least poor	525/1253 (45%; 37–53)	Referent	Referent
**Bednet use**			
Used an ITN the previous night	2188/4127 (56%; 51–61)	0.79 (0.62–0.99)	0.04
Used an untreated net the previous night	227/491 (43%; 32–54)	0.52 (0.34–0.80)	0.003
Did not use a net	1243/2200 (62%; 56–68)	Referent	Referent
**IRS use**			
Lives in a house that received IRS	159/289 (54%; 47–61)	0.63 (0.42–0.93)	0.02
No IRS	3499/6529 (57%; 52–62)	Referent	Referent

a 95% confidence interval.

## Discussion

ITNs have been shown to reduce morbidity and mortality, but coverage continues to be moderate in many parts of sub-Saharan Africa. As much of the malaria control community is shifting away from a narrow strategy of targeted ITN use amongst vulnerable groups such as children <5 years old and pregnant women and towards universal coverage, we explored the gains made through a routine health facility-based distribution system and the potential steps needed to achieve universal coverage. Through the use of health facility-based distribution, Malawi has been able to achieve moderate household ITN possession (59%) and use by all persons (49%), but is still short of universal coverage. However, this distribution strategy is hampered by various factors. First, only 67% of eligible households received an ITN through health facility-based distribution, thus suggesting that this system can only reach a portion of the target population. Although ANC and vaccination clinic attendance is high with 96% of women aged 15–49 years who completed a pregnancy in the past two years attended ANC at least once during their last pregnancy and 97% of children receiving at least one vaccination by 12 months of age [29], this distribution system is still not reaching all eligible pregnant women and children. The efficiency of this system needs to be improved (e.g. decreased ITN stockouts, all women presenting to ANC receiving an ITN) to improve its impact. Second, only 72% of households have a pregnant women or a child <5 years old, thus not all households can be reached by this strategy. However, we found that 36% of households that were ineligible for health facility-based distribution had an ITN obtained from a health facility. This suggests that despite efforts to target ITN distribution, there is a re-distribution of ITNs to the entire population. As countries move towards universal ITN coverage, health facility-based distribution might be an effective means to keep up household ITN possession in between campaigns and might be moderately successful in even reaching ineligible households.

However, despite the moderate success of health facility-based distribution, this strategy is unlikely to lead to universal coverage. We explored the inputs needed (number of ITNs) and the expected coverage achieved by different distribution strategies. Our analysis suggests that universal coverage campaigns that target all households will lead to better distribution of ITNs between households than targeted campaigns that distribute ITNs either to children <5 years old or children 5–15 years old. In addition, a universal coverage campaign to distribute 2 ITNs per household or 1 ITN per sleeping space will require similar inputs and achieve similar coverage. Given the potential difficulty of defining a sleeping space during a distribution campaign, it is likely that a campaign that distributes 2 ITNs per household might be logistically less challenging. A campaign that distributes 1 ITN per 2 people would provide the highest coverage, but would require the largest inputs.

Despite moderate levels of household ITN possession, ITN use among persons who resided in a household with an ITN was high (76%), especially for children <5 years old and pregnant women. Over 92% of all ITNs in the households were hanging at the time of the survey and each net was used by a mean of 2.4 persons, suggesting that concerns that ITNs are not being used are unfounded in this case. Our multivariate logistic regression model suggests that among persons who resided in a household with at least one ITN, use was associated with being in a target group for facility-based free distribution and the number of ITNs per household. Use among all persons increased with increasing number of ITNs per household suggesting that a key barrier to higher ITN use amongst all household members is the lack of ITNs in the household.

Our analysis of ITN possession and use by socioeconomic status suggests two different patterns. Households in the poorer quintiles were less likely to own ITNs despite free distribution through health facilities. This pattern suggests that there might be barriers to persons from poorer households in obtaining ITNs. These barriers might be reduced access to health facilities due to either distance or cost, residing in areas in which health facilities offer poor quality services and have a less functional ITN distribution system, or reduced knowledge about the health facility-based ITN distribution program. However, persons residing in the poorer households were more likely to use the ITNs if they owned them. As children who reside in poorer households have greater disease burden (parasitemia and anemia) than children in less poor households, the potential gains on both reducing inequities in ITN possession as well as disease burden might be substantial if we adopted distribution strategies that are likely to be equitable. Prior equity analyses of ITN distribution campaigns suggest that these campaigns (either universal or targeted) can reduce inequities in ITN possession [3], [4], [7], [13], [15], [30], [31], [32] more quickly and effectively than other strategies.

Although almost all of Malawi experiences malaria transmission, there are marked district level differences in both disease burden and ITN possession and use. The health facility-based ITN distribution program is national, but we noted district-level differences in coverage with ITNs obtained from health facilities. These district level differences might be due to differences in population (e.g. socioeconomic status) as well as the functionality of the health system at the district level. In our analysis we adjusted for socioeconomic status differences, but could not account for other confounders (e.g. education level). District-level differences need to be examined further to better understand potential determinants of district-level health system performance as measured by its ability to deliver key preventive interventions such as ITNs. In addition, measuring and understanding district-level differences might be useful for targeting high disease burden, low coverage districts to achieve maximum coverage with scarce resources.

ITNs have been shown to reduce morbidity and mortality in numerous controlled trials [1]. In our analysis, use of ITNs and untreated bednets by children <5 years old was associated with reduced asexual parasitemia prevalence, a measure of malaria endemicity, and anemia prevalence, a common manifestation of malaria infection that leads to poor health outcomes in children. Thus, we demonstrate a significant disease-specific impact of ITN use in children <5 years old. Given a population of 1,084,969 children <5 years old of a total population of 4,339,876 in the eight districts based on the 2008 census, current levels of ITN use are associated with an estimated 33,908 fewer children with asymptomatic parasitemia and 36,384 fewer children with anemia, and if universal coverage was achieved with all children sleeping under ITN then we can expect 55,587 fewer children with parasitemia and 59,646 fewer children with anemia. In our analysis, both untreated bednets and ITNs had a similar association with reduced parasitemia and anemia prevalence; this finding needs to be explored further.

As we aim for universal coverage, we need to understand both the distribution of ITNs between households as well as within households. In this analysis from Malawi, we show the impact of a particular ITN distribution strategy on achieving household ownership and how this translates into ITN use by individual household members. An understanding of these dynamics is critical to evaluate current distribution programs as well as design future distribution strategies. These types of analyses as well as reporting of both household ITN possession (already done by most major surveys such as the Malaria Indicator Survey (MIS) and Demographic and Health Surveys (DHS)) as well as ITN use by all household members (not currently done by most major surveys) are critical in monitoring our progress towards universal coverage and identifying gaps in coverage, such as low use by particular age groups (e.g. in Malawi, low use by children 5–15 years old).

### Limitations

This study has a number of limitations. We report a survey from 8 of 28 districts in Malawi. Although, these districts contain 33% of the population of Malawi and are geographically diverse, these districts were purposively selected as sentinel districts and thus our findings are not representative of all of Malawi. As with all survey data, the findings are limited by recall and social desirability biases. However, the recall period for most questions was relatively short (e.g. the previous night) and many aspects of ITN possession (e.g. possession of net and its location in the household) and socioeconomic status (e.g. possession of assets and house construction variables) were confirmed by visual inspection.

### Conclusions

Effective vector control through the use of ITNs is one strategy to reduce the substantial malaria burden in Malawi. ANC and vaccination clinic based distribution of ITNs has increased household ITN possession to moderate levels, but falls short of universal coverage. Universal coverage mass distribution campaigns will be needed to achieve maximal public health impact. A successful ‘keep up’ distribution strategy needs to be supplemented with periodic ‘catch up’ campaigns.
